# Using English medium instruction to teach a general course in a College of Business and Management

**DOI:** 10.3389/fpsyg.2022.984869

**Published:** 2022-10-11

**Authors:** Iman Oraif, Mohammed Alrashed

**Affiliations:** Department of English Language and Literature, College of Languages and Translation, Imam Mohammad Ibn Saud Islamic University (IMSIU), Riyadh, Saudi Arabia

**Keywords:** EFL students, ESP, EMI, KSA, higher education

## Abstract

Even though learning can be complex when using a language other than one’s mother tongue, the English-medium communicative approach has been widely adopted in Saudi universities over recent years. Many universities in Saudi Arabia are teaching their courses through the medium of English rather than Arabic, as was previously the case. This also applies to the social and natural sciences. Moreover, instructors are trying to avoid using any Arabic in class, given that English has become the *lingua franca* in many domains. Thus, the current study seeks to understand how students in the College of Business and Management at Al-Imam University, Riyadh, Saudi Arabia feel about English being used as the medium of instruction (EMI) as they study general course. Also examined is the relationship between their previous language test scores and current reflections. Twenty-four female students participated in a survey adapted from generating results to reveal a positive attitude to using EMI on their course. However, no relationship was found between the participants’ language assessment test scores and current feelings.

## Introduction

English is a universal language that is used all over the globe. Therefore, it has become crucial for many to learn English, regardless of their age, profession or status. English is also the language of academia in universities and other institutes of higher learning (Wanphet and Tantawy, 2018). Hence, English-medium instruction (EMI) is a pervasive educational movement, especially in higher-education institutions (Macaro et al., 2018).

Hence, although there are still a number of implementation problems and constraints, EMI has been extensively provided in an increasing number of universities and other institutions worldwide (Earls, 2016). This term refers to employing English as the medium of instruction for various academic disciplines—other than English as a subject in itself—in countries where English is not the native language (Macaro et al., 2018).

The reasons why second language (L2) learners favour EMI programmes are varied and context dependent. However, one important reason is that there is significant demand to incorporate EMI into higher-education content programmes, since it is prestigious to use English to keep track with the latest developments and needs of the marketplace (Knight, 2013). Consequently, the spread of EMI programmes is thoroughly ingrained in educational, economic, scientific and technical developments, as well as in fostering intercultural communication, resulting in the proliferation of EMI programmes in education (Doiz et al., 2013). Additionally, the expansion of English as an international language (EIL) drives competition between individuals and organisations to provide or undertake EMI programmes (Macaro et al., 2018).

Nevertheless, pedagogical models that integrate language learning and content have deep roots in the history of L2 instruction, even prior to the recent global wave of EMI instruction. Wächter and Maiworm (2014) note a 10-fold expansion in EMI programmes in universities across Europe over a 13-year period. Meanwhile, Asian universities have been on the same track, with the tangible development of EMI provision (Fenton-Smith et al., 2017). This fact is supported by Chapple (2015), who found a significant impact of EMI programmes on improving teaching quality to overcome learning barriers. Employing EMI in content programmes offers a means of preparing a workforce that is proficient in English, in order to compete in local as well as the global labour markets (Troudi, 2009).

In terms of the use of EMI in higher education, Saudi Arabia is no exception, with the number of EMI degree programmes growing rapidly in its universities over the past few years. This represents a new trend in an education system that was already moving towards internationalisation. More and more educational institutions have integrated EMI into their curricula in the Kingdom, with the aim of fulfilling the increasing demand for English language skills, including in academia, where English dominates. One aspect of growth is the emergence of EMI in all fields within the education sector; this is particularly evident in higher education and ESP settings.

The increasing number of English as foreign language (EFL) learners in Saudi universities has created an EMI educational context. As such, a major endeavour of several higher-education institutions in Saudi Arabia is the adoption of EMI for language study programmes. There is also a need to explore the use of EMI in ESP university classes in Saudi Arabia. The adoption of EMI in Saudi universities is the main channel for equipping ESP students with content and language proficiency. This enables Saudi higher-education institutions to prepare efficient and competitive ESP graduates for the Saudi and global labour markets. This current study belongs to a limited body of research investigating the current situation involving EMI in Saudi universities, thereby answering the following questions:

1. How do students feel about using English as the medium of instruction (EMI) on their general Management and Business course?
2. What is the relationship between students’ language assessment test scores and their feelings about using EMI on their general course?

## Literature review

### Factors and challenges of implementing English medium instruction

In the EMI literature, there is a basic assumption about the self-perception that L2 learners risk falling behind in their required L2 proficiency (Wächter, 2008; Huang, 2015). The literature highlights two main challenges facing EMI learners in achieving L2 proficiency: the methodology used and the available resources (Garcia, 2020). Similarly, Bradford (2016) proposes the following four types of challenge in the field of EMI: (1) linguistic, (2) cultural, (3) structural and (4) identity-related (institutional).

Linguistic challenges refer to language-related issues that instructors and learners encounter on EMI programmes. Instructors who use EMI give due concern to the inherent linguistic challenges in the heterogeneity of varying levels of language proficiency amongst L2 learners. The main linguistic challenge encountered by instructors is their ability to tackle this diversity, as well as their mastery of the L2 itself. Non-native learners often have difficulty with these issues, as they work hard to comprehend native instructors’ accented English (Ammon and McConnell, 2002). They also encounter difficulty in understanding lecture content that is delivered *via* EMI (Hellekjær, 2010). Additionally, these learners encounter challenges in EMI, due to being unable to fully grasp the academic literature in English as a result of their lack of English language proficiency. Furthermore, Wilkinson (2013) refers to learners at university having problems maintaining EMI programmes, due to their lack of English language proficiency.

Cultural challenges further highlight the mismatch between the attributes and goals of learners outside the country and those within it (Bradford, 2016). For example, the EMI instructor’s experience will have a significant impact on the cultural challenges. For example, a British instructor who applies a learner-centred style of instruction, based mainly on interaction, could find that this teaching style is unfamiliar to L2 university students, who tend to be passive in their learning (King, 2013). Cultural anxiety is another related challenge. This occurs on EMI programmes where there is an associated and apparent superiority attached to teaching English, as opposed to the native language. There is little evidence that EMI has this negative cultural impact (Jenkins, 2013), but the challenge is tangible and needs to be considered when implementing EMI in L2 contexts.

Structural challenges in EMI classrooms are associated with overall coherence and the issues represented in the inadequate number of EMI programmes and support staff, which cannot fulfil the needs of a diverse population (Bradford, 2016). Research has pinpointed the potential reluctance of EMI instructors because of their lack of confidence, stemming from a lack of training or the absence of financial incentives (Byun et al., 2011). As regards the lack of confidence, an overlap can occur with linguistic challenges. Here, the views of administrators and potential EMI instructors could differ over the proposal that distinguished levels of proficiency are necessary to teach EMI courses, whereas there is little or no institutional support to attain those levels. Finally, identity-related (institutional) challenges are associated with the way that the identity of EMI programmes is perceived by the stakeholders, the instructors teaching on those EMI programmes and the learners’ enrolled on them (Bradford, 2016). In addition, these challenges cover the instructors’ views of their identities and status in the workplace, and the significant efforts made by the learners to develop and maintain these identities.

There are significant issues involved in aggravating the cultural challenges facing students, for example, poor or non-existent interaction between international and L2 learners. These same questions and worries about issues such as the faculty’s self-perceptions, and how institutions wish to be seen by the outside world, have been reported in contexts worldwide, for instance, in Malaysia (Ali, 2013), Korea (Byun et al., 2011), and China (Deem et al., 2008). As such, identity is an important issue when implementing EMI in higher education.

### Use of English as the medium of instruction in an L2 context

Researchers have examined EMI in various L2 contexts, portraying a thorough grasp of EMI implementation and learning outcomes. To illustrate this, Dearden (2014) collected data on EMI as an influential, educational trend in 55 countries, identifying the magnitude, distribution, and application of EMI programmes worldwide. Sixty staff members employed by the British Council participated in the above study. The results showed that the respondents favoured EMI and affirmed its extensive growth. Concomitantly, it was found that overall; there was official support for EMI programmes on the part of governments. However, public opinion was only halfway in favour of EMI, reflecting controversial attitudes to such programmes and any plans to expand them. These conflicting attitudes may be attributed to the view that access to EMI programmes is limited by the learner’s socio-economic status, in addition to the apprehension that a learner’s L1 and/or national identity may be diminished by the prevalence of EMI.

**Figure 1:**
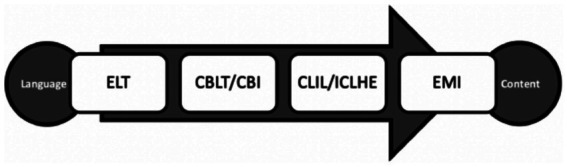
Approaches to language and content teaching (Galloway and Ruegg, 2020).

In many contexts, EMI concurs with governmental goals to boost L2 proficiency. Therefore, EMI has become tantamount to “Englishisation” in various universities worldwide (Curle et al., 2020). The pressing need to develop English language performance is a key motivation for learners to enrol on EMI programmes (Galloway and Ruegg, 2020). This broad expansion in EMI across institutions will continue to proliferate and be adopted by L2 instructors in all educational settings, as there is accelerated demand for academic, professional and technical content to be taught *via* EMI amongst vast numbers of learners. This will promote the use English for communication. An area of this high demand is the deployment of EMI in English for special purposes (ESP) settings (Figure 1).

Ramiro et al. (2015) examined the challenges of EMI in writing classes, in a Spanish study conducted at the University of Almería. It was consequently revealed that the university students experienced difficulty with the cognitive processes that are inherent to comprehending written texts. These difficulties were further aggravated when written tasks were attempted in a non-native language. Hence, the above researchers analysed the written production of university ESP students with an EMI approach. The results highlighted the differences in certain areas of the students’ written performance. There was an evident need to adopt effective methodologies to resolve the difficulties encountered by the students, with the aim of enabling features of L2 writing to be incorporated into their course content.

In another previous study (Wong, 2010); longitudinal action research was conducted to investigate the use of EMI in Hong Kong, comparing the preferences of two EFL classes where two different policies were implemented. In one class, no Cantonese was permitted, whereas in the other, Cantonese was used. The survey results showed that the English-only class was more appreciative of the policy than the class where Cantonese was permitted under a different policy. Similarly, Galloway (2017) examined the role of EMI in universities in both Japan and China. The underlying belief is that EMI will boost university students’ English-language proficiency to produce a workforce that is fluent in English. Thus, there are two related benefits of EMI: content knowledge and L2 mastery. As such, institutions and learners are of the view that EMI can offer more value in the global labour market (Galloway, 2017). However, Galloway found in the above study that the learners grasped more content when studying in their native language than they did using EMI. Their instructors also thought that EMI programmes should adhere strictly to an English-only policy, whereas others have stated that the students’ L1 may be employed as a learning tool on an EMI programme, with EMI serving as a means of delivering course content rather than learning the language itself.

Conversely, Tang (2021) investigated instructors’ views concerning the challenges of teaching EMI, and its outcomes for students’ learning in Thailand International College. The above researcher adopted a qualitative research design, conducting interviews with the study participants. Twelve instructors from four programmes volunteered to be interviewed *via* a purposive sampling procedure. In analysing the interview transcripts, the researcher employed thematic analysis to identify common themes. The results revealed four types of challenge: (1) linguistic, (2) cultural, (3) structural, and (4) identity. In addition, four important conditions were revealed as significant when implementing an EMI programme: (1) improving language, (3) learning subject content, (3) preparing for a career, and (4) working towards internationalisation.

Meanwhile, a more general study was conducted by Ernawati et al. (2021), who undertook a qualitative study to examine the views of Indonesian elementary school teachers and their students, regarding EMI in Paramount School, Palembang. A case study design was employed. The researchers adopted a qualitative research design and interviewed three Science teachers, two Mathematics teachers and 12 students. Thematic analysis was carried out to process the data, and the themes were subsequently coded and classified into distinct types, in order to identify the main themes in light of the participants’ views. The results displayed similarity in the instructors’ and students’ views of EMI in the classroom. Overall, the instructors and students had positive views of EMI, due to its benefits.

According to the conclusions drawn in previous studies, students who are taught general English course tend to dislike the idea of EMI. Thus, this current study sought to investigate the situation in a Saudi university, looking at the use of EMI on a general English course at the College of Business and Management.

## Research methodology

### Study design

This study follows a quantitative approach, using surveys. The data were collected through purposive sampling, with a class being selected from the two available classes at the College of Business and Management in a university in the Kingdom of Saudi Arabia (KSA). The English course included the teaching of four skills, with vocabulary relating to management and administration. The course was designed to enable learners to practice real-life communication in the work context. The textbooks used are *designed for general courses in Management and Business.* One example of the topics covered is production.

The course was taught over a period of 15 weeks, with three face-to-face (F2F) classes weekly, each of 12 h’ duration. The teacher used enrichments like YouTube videos for certain difficult topics in the curriculum, like using the past tense in English. At the end of term, the learners gave a presentation about a topic related to their course, drawn from their textbook. The students were divided into groups of five to deliver these presentations, whereupon they shared the collected data. Each student presented for 5 min.

While teaching, the tutor only used English, without resorting to Arabic at any time. She even provided help through the medium of English. In this study, the teacher was the researcher, but she had no influence on the participants’ opinions, and the students’ participation was purely voluntary. The data were collected at the end of term, after the learners had received their final grades. This would ensure that their participation did not impact their actual grades. Oral permission to gather the data was granted by the Head of the Department.

The survey was adapted from Wong (2010), replacing Cantonese with Arabic, this being the participants’ L1. The above survey was originally constructed to measure the effectiveness of using Cantonese as the sole medium of instruction at a secondary school in Hong Kong. The previous research adopted an action research approach, whereas this present research consisted of a case study. Wong (2010) also used pre-and post-language tests, with interviews. The original scale for measuring the survey responses consisted of a six-point Likert scale (with answers ranging from 6 = Strongly agree to 1 = Strongly Disagree, with 5 = Agree; 4 = Tend to agree; 3 = Tend to Disagree and 2 = Disagree). However, the present researcher adjusted this scale to a four-point instrument, making it easier for the participants to decide on their responses. Likewise, the survey was translated into Arabic to facilitate the participants’ understanding and encourage them to respond. The demographic data were gathered using open-and closed-ended questions.

Reliability is a very important factor to consider when selecting a questionnaire instrument. Reliability refers to the degree of consistency or stability in a study’s results if it is conducted using the same respondents on several occasions. In order to evaluate reliability, the Cronbach’s alpha values were calculated for each dimension in this study. Cronbach’s alpha (α), developed by psychologist Lee Cronbach in 1951, is the most common estimate of reliability. It is based on the inter-correlation between the observed indicator variables. Cronbach’s alpha produces values of between 0 and 1, and its acceptable range is between 0.7 and 1.

Table 1 demonstrates that the data passed the reliability test, as all Cronbach’s alpha values exceeded the acceptable value. For the questionnaire, the Cronbach’s alpha value was 0.783 (greater than 0.7).

**Table 1:** Reliability analysis.

	**No. of Items**	**Cronbach’s Alpha**
TOTAL	11	0.796

### Study population

#### Characteristics of the sample

This section presents the main characteristics of the sample, which comprised 24 respondents out of the 32 students who received the questionnaire. Only female participants were involved because of religious gender restrictions in the study context. However, different ages and university levels were represented in the sample. The following Tables display the participants’ personal information (i.e. university level, specialisation, age and whether they had taken any English language assessment test to measure their English language level). From the results, it can be seen that the participants were homogeneous.

From the data in Table 2, above, it is clear that 91.7% of the students were in the first level of university, whereas 8.3% were in the second level. This shows that the vast majority of the sampled students were in the first university level.

**Table 2:** Table of frequency for university level.

	**Frequency**	**Percentage**
First level	22	91.7
Second level	2	8.3
Total	24	100.0

From the data in Table 3, above, it is clear that the ratio for Economics was 62.5% and the ratio for Management was 37.5%. Both specialties are taught in the College of Business and Management and topics in the used books are related to these specialties.

**Table 3:** Table of frequency for specialisation.

	**Frequency**	**Percentage**
Management	9	37.5
Economics	15	62.5
Total	24	100.0

Table 4 demonstrates that 83.3% of the sampled participants were aged between 18 and 21 years, while 16.7% of the sample was aged between 21 and 24 years, which indicates that they are homogeneous in their age.

**Table 4:** Table of frequency for age.

	**Frequency**	**Percentage**
18–21 years	20	83.3
21–24 years	4	16.7
Total	24	100.0

From the data in Table 5, above, it is clear that the students who had taken a test to measure their English language level amounted to 37.5%, while those who had never taken such a test totalled 62.5%.

**Table 5:** Table of frequency for students having taken a test to measure their English language level.

	**Frequency**	**Percentage**
Yes	9	37.5
No	15	62.5
Total	24	100.0

## Results

The following sections present a statistical analysis of the questionnaire responses. The aim of this study is to investigate the following: How do students feel about using English as the medium of instruction (EMI) on their general Management and Business course? What is the relationship between students’ language assessment test scores and their feelings about using EMI on their general course?

In this part of the study, statistical data analysis was conducted as follows: the main sample characteristics were first presented, describing the participants’ personal information (i.e. university level, specialisation, age and whether the student has ever taken a test to measure her English language level). Reliability analysis was subsequently conducted, before presenting the results of the hypothesis-testing.

## Hypotheses-testing

The following Table (Table 6) presents the participants’ opinions of an English language lecture delivered in English (for English 1).

**Table 6:** Mean and standard deviation (SD) for students’ opinions of having English as the medium of instruction on their Business English course.

**No.**	**Items**	**Mean**	**SD**	**%**	**Ranking**	**Response**
11	As these are English lessons, we should use English as much as possible.	3.50	0.590	88%	1	Strongly agree
8	I would not be upset if my friends also spoke English in class.	3.46	0.509	86%	2	Strongly agree
7	It is fun to listen to my friends speaking English in class.	3.38	0.576	84%	3	Strongly agree
2	Only using English in the classroom could improve my standard of English.	3.25	0.737	81%	4	Strongly agree
6	Enforcing the ‘No Arabic in English Class’ policy does not alarm me.	3.21	0.833	80%	5	Agree
1	I like it when my English teacher only uses English as the medium of instruction.	3.17	0.761	79%	6	Agree
5	English teachers are good examples if they only use English in an English class.	3.17	0.761	79%	7	Agree
4	Given the choice, I prefer using English only in English classes instead of a mixture of English and Arabic.	3.04	0.806	76%	8	Agree
3	My confidence in speaking English has been increased.	2.79	0.779	70%	9	Agree
10	If my English teacher uses Arabic to teach English, I will be annoyed.	2.38	0.970	59%	10	Do not agree
9	If my friends do not speak English, I will not either.	2.21	0.833	55%	11	Do not agree
	Total	3.05	0.432	76%		Agree

### How do the students feel about using English as the medium of instruction on their general management and business course?

The following Table presents the participants’ opinions and feelings about English being used as the medium of instruction on their Business English course.

It can be seen from Table 6 that the students’ feelings about English being used as the medium of instruction on their Business English course were highly positive, meaning that the students largely preferred learning through EMI, with a mean of 3.05 and percentage of 76%.

Item 11 were the most frequently indicated, stating: “As these are English lessons, we should use English as much as possible.” The students “Strongly agreed” with this, resulting in a mean of 3.50, SD of 0.590, and percentage of 88%. The second most frequently indicated was Item 8, with which the students also “Strongly agreed.” This Item stated: “I would not be upset if my friends also spoke English in class,” resulting in a mean of 3.46, SD of 0.509 and percentage of 86%. In descending order of frequency, the rate of response for the remaining items was as follows. The students “Strongly agreed” with Item 7, which stated: “It is fun to listen to my friends speaking English in class,” resulting in a mean of 3.38, SD of 0.576 and percentage of 84%. However, the students “Did not agree” with Item 10, which stated: “If my English teacher uses Arabic to teach English, I will be annoyed,” resulting in a mean of 2.38, SD of 0.970 and percentage of 59%. Likewise, the students “Did not agree” with Item 9, which stated: “If my friends do not speak English, I will not either,” resulting in a mean of 2.21, SD of 0.833 and percentage of 55%.

### What is the relationship between the students’ language assessment test scores and their feelings about using English as the medium of instruction on their general course?

To discover the relationship between the students’ English assessment test scores and their responses concerning the use of EMI on their Business English course, an independent sample *t*-test and Pearson’s correlation coefficient test were conducted.

It is clear from Table 7, above, that there was no statistically significant relationship at the 0.05 level between the students’ scores in their English assessment tests and responses concerning the use of EMI on their Business English course (*p* > 0.05).

**Table 7:** Independent sample *t*-test and Pearson’s correlation coefficient test results.

**Have you ever taken a test to measure your level of English?**	**No.**	**Mean**	***SD***	***t***	**df**	***P***	***R***	***P***
Yes	9	3.20	0.535	1.360	22	0.188	0.287	0.188
No	15	2.96	0.345					

## Discussion

As the results indicate, the use of EMI was preferred by most of the participants. The participants strongly agreed that English should be used as much as possible, since it was an English class. The provision of more language input in class, specifically from a trusted individual – like their teacher – can help develop students’ language skills. An input hypothesis developed by Krashen (1992) states that we acquire language when we understand the message delivered or where the input is comprehensible. Abukhattala (2013) also adds that learning a language under this hypothesis is like learning how to drive a car, where you acquire the knowledge then start to drive by yourself.

According to the input hypothesis, learners go through a silent period after being provided with comprehensible input. The duration of this period will differ from one person to another. However, the learners will grow in confidence and start producing the language. Hence, such a hypothesis is more applicable to productive skills like writing or speaking. In that sense, introducing learners to the language extensively through EMI can help provide more comprehensible input, so they are more likely to produce the language. This can also be related to the participants’ responses, where they strongly agreed that using only English in class could help them improve their standard of English.

The participants also mentioned that they had fun listening to their friends speaking English. Enjoyment and fun can play an essential role in learning a foreign language. Moreover, it can lower anxiety amongst learners about responding to questions and using the language. Anxiety can prevent successful language learning, especially in the productive skills, like speaking. In a review study conducted by Hanifa (2018, p.1), the researcher found that the possible causes of anxiety about speaking were (1) cognitive factors, topics, genre, interlocutors, and processing demands; (2) affective factors dealing with feelings about a topic and/or the participants, and self-consciousness, and (3) performance factors, concerning the mode, degree of collaboration, discourse control, planning and rehearsal time, time pressure and environmental conditions. The study concluded with the recommendation that understanding the source of anxiety would help the teacher identify the reasons and gain more insights into finding solutions to lower anxiety. Thus, allowing learners to use L2 amongst themselves, as the current study shows, could play a crucial role in reducing their anxiety.

Furthermore, in this present study, the participants agreed with the statement that their self-confidence had increased through EMI. Self-confidence plays a vital role in improving language proficiency. In a study conducted by Jabor et al. (2017), within a similar context to the current study, the researchers collected data on self-confidence levels using a questionnaire. They found that the female students displayed low self-confidence in speaking English with their classmates and professors. The study also suggested adopting a suitable teaching approach to urge students to use the English language and overcome their social and psychological obstacles. Hence using EMI can help as being indicated in the current study results.

Meanwhile, the participants found that using Arabic (their L1) in their class would not annoy them. However, use of their L1 should be limited to the most problematic elements in the syllabus, which the learners found hard to acquire. This result corresponds to the outcome of a study by Alshehri (2017), who explored the attitudes of EFL teachers with regard to using the learners’ L1 in EFL classes. The study followed a mixed methods approach, implementing questionnaires and follow-up interviews to collect data from EFL preparatory year teachers in a state university in Saudi Arabia. The results reflected that the teachers found L1 use to be helpful to some extent, serving pedagogical functions such as explaining vocabulary. The current study also supported this idea about using the L1, finding no relationship between the language level assessment test and the participants’ attitudes to EMI. Therefore, some learners will avail themselves of the available support by using their L1.

## Conclusion

The use of English as a medium of instruction or EMI has not been common in many specialties in the universities in Saudi Arabia. This is despite the fact that the current transition towards an English language curriculum, rather than using the students’ L1, necessitates further research about EMI. In KSA, there is a dearth of research on the attitudes and acceptance of learners and teachers regarding EMI. This is especially true since most Saudi learners are not at a sufficient level of English language proficiency to study content in English; instead, they need to develop their language level. First, the students themselves may be a factor. The other main factor is the instructors’ language ability, which must be assessed to ensure a successful transition. This study presents positive results of using EMI in a general course in the College of Economics and Administration, but it encourages more research in this area in the context of Saudi higher education.

## Data availability statement

The original contributions presented in the study are included in the article/supplementary material, further inquiries can be directed to the corresponding author.

## Author contributions

IO and MA contributed to different parts in the research. All authors contributed to the article and approved the submitted version.

## Conflict of interest

The authors declare that the research was conducted in the absence of any commercial or financial relationships that could be construed as a potential conflict of interest.

## Publisher’s note

All claims expressed in this article are solely those of the authors and do not necessarily represent those of their affiliated organizations, or those of the publisher, the editors and the reviewers. Any product that may be evaluated in this article, or claim that may be made by its manufacturer, is not guaranteed or endorsed by the publisher.
